# The Complete Mitochondrial Genome of Red Costate Tiger Moth (*Aloa lactinea* [Cramer, 1777]), and Phylogenetic Analyses of the Subfamily Arctiinae

**DOI:** 10.3390/genes16050554

**Published:** 2025-04-30

**Authors:** Chengrong Pan, Sheng Xu, Yu Shu, Jie Fang

**Affiliations:** 1 Anhui Province Eco-Environmental Monitoring Center, Hefei 230071, China; ccrp@sina.com (C.P.);; 2 College of Life Sciences, Anhui University, Hefei 230093, China

**Keywords:** *Aloa lactinea*, Arctiinae, Lepidoptera, mitochondrial whole genome, phylogenetic analysis

## Abstract

Background/Objectives: *Aloa lactinea*, class Insecta, order Lepidoptera, superfamily Noctuoidea, family Erebidae, and subfamily Arctiinae, is a polytrophic agricultural pest. However, there are still many sequences missing for Arctiinae from mitochondrial whole-genome sequences. Methods: In this study, we determined and analyzed the complete mitochondrial genome sequence of *A. lactinea*. Furthermore, based on the sequencing results, we used the Bayesian inference, maximum likelihood, and maximum reduction methods to analyze the phylogenies of 18 species of the Hypophora subfamily. Results: The mitochondrial genome was found to be a circular double-stranded DNA with a length of 15,380 bp and included 13 protein-coding genes (PCG_S_), 22 tRNA genes, 2 rRNA genes, and one control region. With the exception of *tRNA^Ser(AGC)^*, all the tRNA genes could form conventional clover structures. There were 23 intergenic spacer regions with lengths of 1–52 bp and six gene overlaps with lengths of 1–8 bp. The control region was located between *rrnS* and *tRNA^Met^* genes and comprised 303 bp and an AT content of 74.25%. Conclusions: The results showed that *A. lactinea* is closely related to *Hyphantria cunea*. Our results suggest that Syntomini is phylogenetically distinct from Arctiini and may warrant separate tribal status within Arctiinae. This study is dedicated to researching the mitochondrial genome and phylogenetic relationships of *A. lactinea*, providing a molecular basis for its classification.

## 1. Introduction

The red costate tiger moth (*Aloa lactinea*) belongs to the Arctiinae subfamily, Noctuoidea superfamily, Erebidae family, and Lepidoptera order. It is mainly found in China, Japan, Korea, India, Vietnam, Myanmar, Nepal, Sri Lanka, Indonesia, and other Asian countries [1,2]. The most distinctive feature of the adult *A. lactinea* is the presence of a distinct red band on the anterior edge of its white wings, and the unique pattern of alternating orange-yellow and black transverse bands on the dorsal side of its abdomen. The male moths have black serrated antennae, red-tipped black labial palps, and red-black feet. These combined features can serve as reliable identification criteria [3]. Hundreds of plants serve as hosts for red costate tiger moths, such as Cruciferae crops, beans, green onions, and other vegetables, making them notable polytrophic agricultural pests [1,2]. The larvae of the red costate tiger moth are voracious, feeding on the stems, flowers, and fruits of host plants. In severe cases, they may defoliate entire plants, leaving only veins and flower stalks [4]. Their eggs tend to be clustered, and larvae disperse after the third instar, chewing holes into leaves.

The taxonomic status of Noctuoidea has undergone continuous revision, and the phylogenetic relationships within this superfamily remain a subject of debate [5]. Initially, Miller proposed that Noctuidae comprised seven subfamilies (Oenosandridae, Doidae, Notodontidae, Lymantriidae, Arctiidae, Aganaidae, and Noctuidae) [6]. However, Scoble contended that Aganainae should be classified as a subfamily within Noctuidae. Subsequently, with advancements in molecular biology techniques, Lafontaine and Fibiger suggested that Noctuidae consists of five subfamilies (Oenosandridae, Doidae, Notodontidae, Micronoctuidae, and Noctuidae) [7]. Zahiri et al., based on recent morphological and molecular analyses [5], proposed a revised classification for Noctuoidea, which offers hope for a more stable family-level taxonomy. Within Noctuoidea, the subfamily Arctiinae includes over 11,000 species [8]. Due to significant morphological similarities and genetic diversity, classifying genera and species within Arctiinae remains particularly challenging, especially for groups that diverged relatively recently [9]. To date, the systematic classification of species within Arctiinae remains unresolved.

Mitochondria are semi-autonomous organelles with independent genetic material in eukaryotic cells [10]. The mitochondrial genome has strict maternal inheritance, is an ideal molecular genetic marker, and has been widely used for species identification in population genetics, molecular diagnoses, and phylogenetic studies [11]. The mitochondrial genome of Lepidoptera is a circular double-stranded DNA molecule of 15–16 kb [12]. Because of their high mutation rates, rapid evolution rates, and ease of expansion, mitochondrial genomes are widely used in species classification and phylogenetic studies and are important molecular markers [13,14]. However, only 22 whole mitochondrial genome sequences have been determined and assembled, with many missing for Arctiinae [9,15,16,17,18].

In this study, we determined and analyzed the whole mitochondrial genome sequence of *A. lactinea*. We further compared the mitochondrial genome of Rufibridae with those of 17 other species from the subfamily. Based on the mitochondrial genome sequences, we constructed phylogenetic trees of 18 species within the subfamily and discussed the phylogenetic relationships within the subfamily, providing a molecular basis for the classification of various genera and species of the subfamily to lay a foundation for research on the molecular identification, taxonomic status, and mitochondrial genomes of the subfamily.

## 2. Materials and Methods

### 2.1. Sample Collection and DNA Extraction

The two *A. lactinea* specimens used in this study were collected by the authors from Fuxi, Huangshan City, Anhui Province, China (30°09′28″ N, 118°05′54″ E), on 4 July 2023. Four legs were excised from each specimen and preserved in anhydrous ethanol at –20 °C until processing. Mitochondrial genomic DNA was extracted from the ethanol-fixed tissues using the standard phenol/chloroform method [19]. DNA concentration and quality were assessed by 1% agarose gel electrophoresis [20].

### 2.2. DNA Sequencing and Splicing

A starting amount of 1 μg of DNA was used for whole-genome library construction. The extracted DNA was broken down by ultrasound to 300–500 bp. The TruSeqTM Nano DNA Sample Prep Kit (Illumina, San Diego, CA, USA) was used for terminal repair, adding an A tail to the 3′ end, and connecting indexed splices, followed by 8-cycle PCR amplification (50 μL reaction system: 30–40 μL DNA fragments, 5 μL primer mix, 25 μL 2× master mix, and nuclease-free water to adjust the final volume to 50 μL). The PCR protocol was set as follows: initial denaturation at 98 °C for 30 s; 8 cycles of denaturation at 98 °C for 10 s, annealing at 60 °C for 30 s, and extension at 72 °C for 30 s; followed by a final extension at 72 °C for 5 min. The PCR products were size-selected (300–500 bp) via 2% agarose gel electrophoresis, purified, and then subjected to paired-end sequencing (2 × 150 bp) with a sequencing depth of 52× on the Illumina NovaSeq 6000 platform at Beijing Qingke Biotechnology Co., Ltd. (Beijing, China). Raw reads underwent quality control using Trimmomatic v0.39 (http://www.usadellab.org/cms/index.php?page=trimmomatic) (accessed on 1 July 2024) [21], with default settings applied. This quality control process included the removal of adapter sequences, trimming of non-AGCT bases at the 5′ end, exclusion of low-quality bases (Phred score < 20) from read termini, discarding of reads containing more than 10% ambiguous bases (N), and elimination of fragments shorter than 75 bp after trimming. The resulting high-quality reads were deemed appropriate for subsequent analyses. For the PacBio Sequel II platform data, the raw BAM files were converted into FASTQ format through a stringent quality control pipeline. This pipeline included (1) an initial filtering step to discard Polymerase reads shorter than 200 bp, (2) removal of low-quality reads with accuracy scores below 0.80, (3) extraction of Subreads with subsequent trimming of adapter sequences, and (4) a final filtering step to exclude Subreads under 200 bp in length. This comprehensive approach ensured the generation of high-fidelity third-generation sequencing data, ready for downstream genomic applications. Illumina sequencing data were assembled using GetOrganelle v1.7.5 (https://github.com/Kinggerm/GetOrganelle) (accessed on 1 July 2024) [22]. Align the second-generation assembled sequences to the PacBio third-generation data using BWA v0.7.17 [23], and extract the target sample’s third-generation data. Mix the extracted third-generation data with the second-generation data for assembly using SPAdes v3.14.1 [24]. Select sequences with high coverage depth and longer assembly lengths as candidates. Align these sequences to the NT database to identify mitochondrial scaffold sequences, then use overlap to join the sequences. Align the clean reads back to the mitochondrial genome sequence and use Pilon v1.23 [25] to correct the bases. Determine the starting position and orientation of the mitochondrial assembly sequence based on the reference genome to obtain the final mitochondrial genome sequence. Remove redundancy from the initial gene predictions made by MITOS, manually correct the start and stop codon positions, and obtain a high-accuracy set of conserved genes. Use the CGView v2.0.1 software (http://stothard.afns.ualberta.ca/cgview_server/) (accessed on 1 July 2024) [26] to generate a circular map of the sample genome.

### 2.3. Mitochondrial Genome Annotation

Base composition and relative synonymous codon usage were calculated using MEGA v11.0 software [27]. The Mitochondrial Genome Annotation Server (http://mitos.bioinf.uni-leipzig.de/index.py) (accessed on 3 July 2024) [28] was used to predict the protein-coding, tRNA, and rRNA genes from the whole mitochondrial genome. Remove redundancy from the initial gene predictions made by MITOS, manually correct the start and stop codon positions, and obtain a high-accuracy set of conserved genes. Use the CGView software (http://stothard.afns.ualberta.ca/cgview_server/) (accessed on 3 July 2024) [26] to generate a circular map of the sample genome. The tRNA secondary structure was predicted using tRNA structure 5.7 [29]. The secondary RNA structure was calibrated based on predictive models from other insect species [30,31,32,33,34]. Tandem Repeats Finder v4.1software was used to identify tandem repeats in the A+T-rich region [35]. Chain asymmetry was calculated using Formulas (3.1) and (3.2) [36] as follows:

A-T skew = [A% − T%]/[A% + T%]

G-C skew = [G% − C%]/[G% + C%]

### 2.4. Phylogenetic Analysis

In this study, we utilized the newly sequenced complete mitochondrial genome of *A. lactinea* and combined it with sequences from 22 Arctiinae species obtained from GenBank (Table 1) to construct the phylogenetic relationships within the subfamily Arctiinae. Based on their genetic distances, five species—*Eilema depressum*, *Eilema sororculum*, *Cybosia mesomella*, *Spilarctia lutea*, and *Miltochrista miniata*—were selected as outgroups [37].

Complete mitochondrial genome sequences were retrieved from the NCBI database to construct the dataset. Sequence alignment was performed using MAFFT v7.0 [38], followed by manual curation. The dataset was then concatenated using PhyloSuite v1.2.3. Bayesian inference (BI) was performed using MrBayes 3.1.2 [39], with the optimal substitution model (GTR) selected based on jModeltest v2.1 [40]. The BI analysis was initiated with a random starting tree, and the Markov Chain Monte Carlo algorithm was set to four chains (three hot chains and one cold chain), which simultaneously ran 10 million generations. Sampling took place once every 1000 generations, when the mean standard deviation of separation frequency was less than 0.01 and the ESS value in TRACER 1.5 was greater than 200. The first 25% of trees were discarded as Burnin samples, and the remaining samples were used to build a Consensus tree. Bayesian posterior probability (BPP) was then calculated [39,41]. Maximum likelihood and maximum parsimony analyses were conducted in MEGA v11.0 [27], under the same model, with branch support evaluated using the ultrafast bootstrap method (1000 replicates).

**Table 1:** Information of species used for phylogenetic analysis of Arctiinae in this study.

*Genus*	*Species*	Genbank Sequence Number	
*Vamuna*	*Vamuna virilis*	NC_026844.1	\
*Eilema*	*Eilema ussuricum*	MN696172.1	Unpublished
*Cyana*	*Cyana* sp. *MT-2014*	KM244679.1	[42]
*Paraona*	*Paraona staudingeri*	NC_037515.1	\
*Spilosoma*	*Spilosoma lubricipeda*	MK903030.1	Unpublished
*Arctia*	*Arctia plantaginis*	NC_057559.1	[43]
*Hyphantria*	*Hyphantria cunea*	GU592049.1	[30]
*Callimorpha*	*Callimorpha dominula*	NC_027094.1	[44]
*Lemyra*	*Lemyra melli*	NC_026692.1	[33]
*Nyctemera*	*Nyctemera arctata albofasciata*	KM244681.1	[42]
*Nyctemera*	*Nyctemera adversata*	NC_062185.1	Unpublished
*Phragmatobia*	*Phragmatobia fuliginosa*	NC_062183.1	Unpublished
*Spilarctia*	*Spilarctia subcarnea*	KT258909.1	Unpublished
*Spilarctia*	*Spilarctia casigneta*	NC_060594.1	Unpublished
*Pareuchaetes*	*Pareuchaetes insulata*	NC_062088.1	Unpublished
*Amata*	*Amata formosae*	KC513737.1	[31]
*Amerila*	*Amerila alberti*	NC_062176.1	Unpublished
*Aloa*	*Aloa lactinea*	\	This study
*Miltochrista*	*Miltochrista miniata*	OW121779.1	[37]
*Eilema*	*Eilema sororculum*	OU618562.1	\
*Eilema*	*Eilema depressum*	OU612042.1	\
*Cybosia*	*Cybosia mesomella*	OX276419.1	\
*Spilarctia*	*Spilarctia lutea*	OU696502.1	\

## 3. Results

### 3.1. Mitochondrial Whole Genome Basic Structure

**Figure 1:**
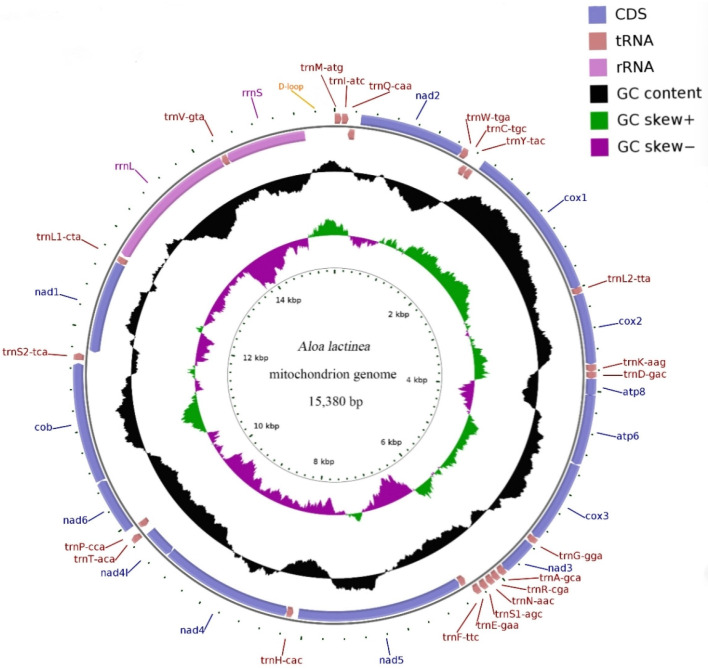
Schematic Map of the complete mitochondrial genome of *Aloa lactinea*. Protein-coding genes (PCGs) are indicated by blue arrows, transfer RNA (tRNA) genes are shown as brown arrows, and ribosomal RNA (rRNA) genes are represented with purple arrows. Each tRNA is annotated using a single-letter amino acid code followed by its specific anticodon. The black circular plot displays GC content, where peaks extending outward indicate values above the average, and those directed inward signify values below the average. GC skew is visualized using purple and green plots: purple represents skew values ranging from 0 to 1, while green indicates values between −1 and 0. Sequence length is marked with ticks along the inner circle.

**Figure 2:**
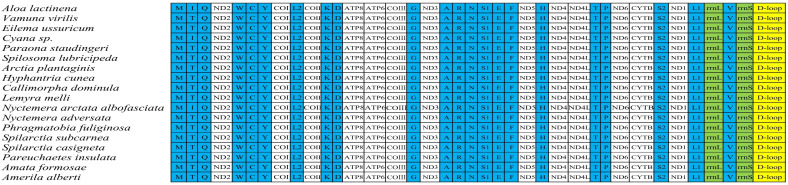
The gene order of the *A. lactinea* mitogenome and the other 18 Arctiinae species (the complete information for the missing five species is unavailable in NCBI). The tRNA genes are represented in blue, the PCGs genes are represented in white, the rRNA genes are represented in green, and a hypothetical control region is represented in yellow.

The mitochondrial genome was observed to be a circular double-stranded DNA of 15,380 bp and included 37 genes and one control region. These 37 genes comprised 13 protein-coding genes, 22 tRNA genes, and 2 rRNA genes. There were 9 protein-coding genes (ND2, COI, COII, ATP8, ATP6, COIII, ND3, ND6, CYTB), 14 tRNA genes (*tRNA^Ala^*, *tRNA^Arg^*, *tRNA^Asn^*, *tRNA^Ser(AGC)^*, *tRNA^Glu^*, *tRNA^Thr^*, *tRNA^Ser(TCA)^*, *tRNA^Met^*, *tRNA^Ile^*, *tRNA^Trp^*, *tRNA^Leu(TTA)^*, *tRNA^Lys^*, *tRNA^Asp^*, *tRNA^Gly^*), and control areas located on the H chain. Eight tRNA genes (*tRNA^Gln^*, *tRNA^Cys^*, *tRNA^Tyr^*, *tRNA^Phe^*, *tRNA^His^*, *tRNA^Pro^*, *tRNA^Leu(CTA)^*, *tRNA^Val^*), four protein-coding genes (ND1, ND5, ND4, ND4L), and two rRNA genes were located on the L chain (Figure 1 and Table 2). The whole mitochondrial genome structure was the same as that of other existing species of Arctiinae (Figure 2).

### 3.2. Nucleotide Composition and Deviation Analysis

**Figure 3:**
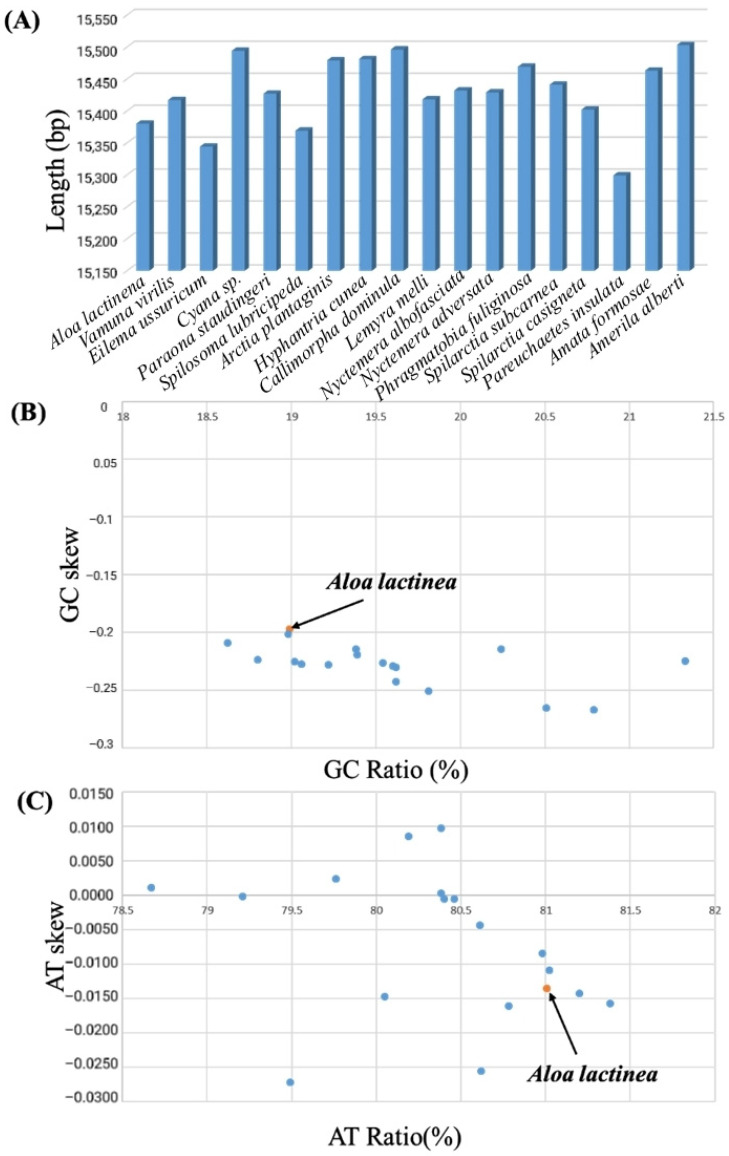
Mitochondrial genome length, AT% vs. AT-Skew, and GC% vs. GC-Skew graphs in Arctiinae mitochondrial genomes. (**A**) The *X*-axis indicates 18 Arctiinae mitochondrial genomes, and the *Y*-axis represents the length of their complete mitochondrial genome. (**B**) The *X*-axis presents the GC ratio, and the *Y*-axis shows GC-skew. (**C**) The *X*-axis presents the AT ratio, and the *Y*-axis shows AT-skew. The orange dotted box indicates *A. lactinea* assembled in this study.

The mitochondrial genome of *A. lactinea* has a nucleotide composition of T (41.05%), A (39.95%), C (11.37%), and G (7.62%), resulting in an A+T content of 81.01% and a G+C content of 18.99%. All genes exhibit a significantly higher A+T content compared to G+C. The A+T content of tRNA genes is 81.87%, while that of rRNA genes reaches 85.28%. The total A+T content of protein-coding genes (PCGs) is 79.92%, with variation among individual PCGs: the lowest A+T content is observed in CO1 (72.64%), and the highest in ATP8 (93.83%). Across the entire genome, all genes consistently show a significantly higher A+T content than G+C. The AT skew values are positive for tRNA and rRNA genes, while other regions exhibit negative values. GC skew values are positive in ND5, ND4, ND4L, and ND1, as well as in tRNA and rRNA genes, whereas other regions show negative GC skew values (Table 3). The nucleotide composition and deviation of the whole mitochondrial genome of *Corymbophora rubra* were consistent with those of other species of Corymbophorinae. *Spilosoma lubricipeda* had the highest A+T content (81.38%) in the mitochondrial genome of the existing species. *Lemyra melli* had the lowest A+T content (78.67%) (Table 4). Both *A. lactinea* and the other 22 Arctiinae species mentioned in this study showed negative GC-skew, but while *A. lactinea* had negative AT-skew, some Arctiinae species displayed positive AT-skew (Figure 3).

### 3.3. The Use of Protein-Coding Genes and Codons

**Figure 4:**
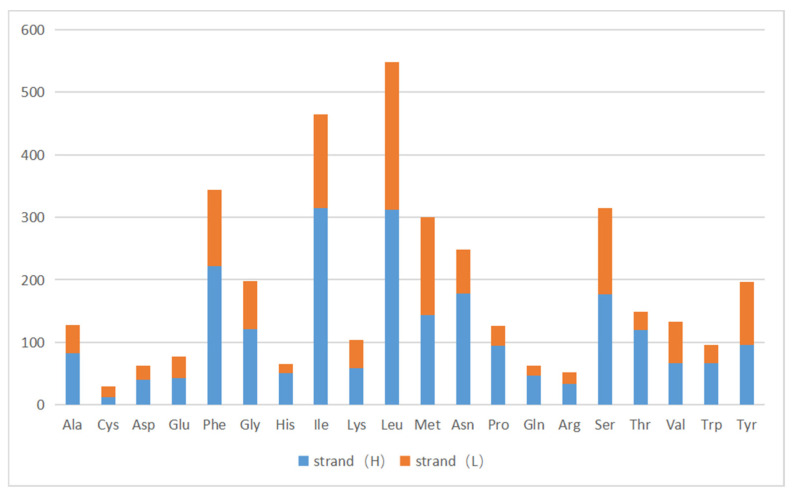
Codon usage of the *A. lactinea* mitochondrial genome. The *X*-axis presents amino acids, and the *Y*-axis presents number of amino acids along with the direction of genes. Strand (H) is the blue color, and strand (L) is the orange color.

**Figure 5:**
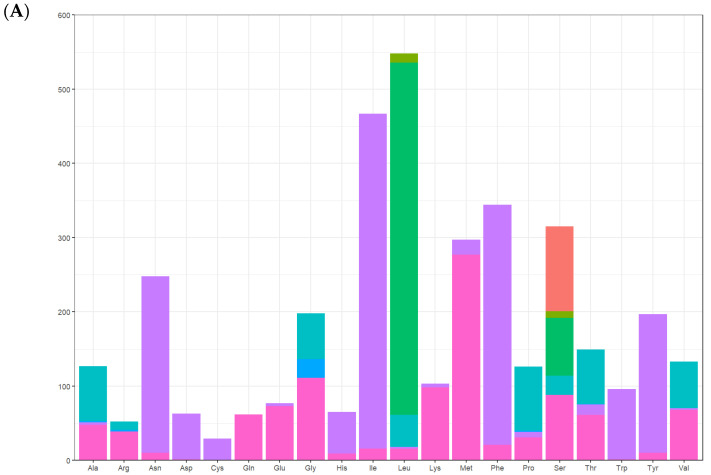

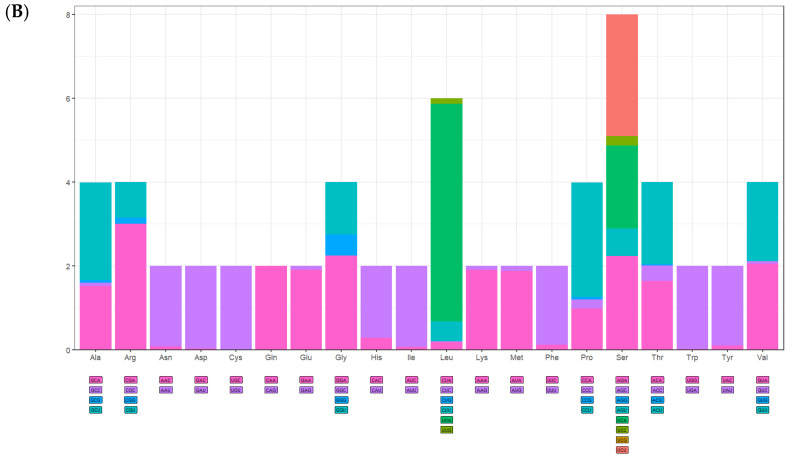
Relative synonymous codon usage (RSCU) of the mitogenomes of *A. lactinea*. (**A**) The *X*-axis presents amino acids, and the *Y*-axis presents number of amino acids. (**B**) The *X*-axis presents amino acids, and the *Y*-axis presents RSCU.

There were 13 protein-coding genes, of which 9 were on the outer ring H chain and 4 were on the inner ring L chain (Figure 1 and Table 2). The number of amino acids represented by the codons in the outer ring H chain was significantly greater than that in the inner ring L chain (Figure 4). ND2, COI, and ATP8 used ATT as their start codon; COII, ATP6, COIII, ND4, CYTB, and ND1 used ATG as their start codon; and ND3, ND5, ND4L, and *ND6* used ATA as their start codon. Except for the incomplete stop codon T of COII and ND4, the other protein-coding genes used TAA as the stop codon. Except for tryptophan (Trp), which uses only the TGG codon, the most frequently used amino acid codons were NNT or NNA. In addition to arginine (Arg), glutamine (Gln), glutamic acid (Glu), glycine (Gly), leucine (Leu), and valine (Val), codons with the highest frequency of use strictly corresponded to tRNA anticodons, and codons with the highest frequency of use did not correspond to tRNA anticodons (Table 5). The 13 protein-coding genes showed a high AT preference (Table 3). In the RSCU analysis, the top three most frequently used codons were UUA (frequency F = 12.85%, RSCU = 5.20), CGA (frequency F = 1.06%, RSCU = 3.00), and UCU (frequency F = 3.08%, RSCU = 2.90), encoding Leu, Arg, and Ser, respectively (Table 5, Figure 5).

### 3.4. rRNA and tRNA Genes

**Figure 6:**
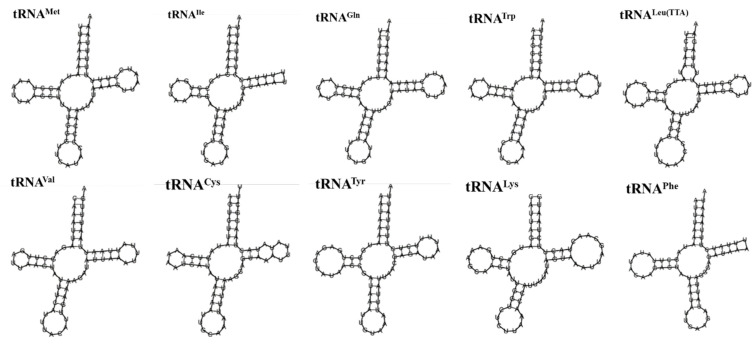

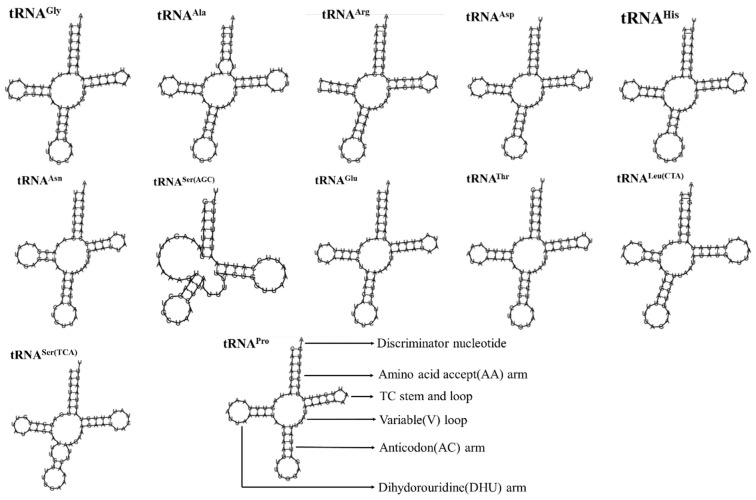
The secondary structure predictions of tRNA genes of *A. lactinea*.

As shown in Table 2 and Figure 6, there were 22 tRNA genes in the mitochondrial genome of *A. lactinea*, with an average length of 66 bp; the shortest one was the *tRNA^Arg^* gene, with a length of 63 bp, and the longest one was the *tRNA^Lys^* gene, with an A+T content of 81.87%, indicating an obvious AT bias (Table 2). Except for *tRNA^Ser(AGC)^*, all the tRNA genes could form conventional clover structures. A total of 16 mismatched base pairs were identified in the tRNA, all of which were U-G mismatched base pairs and existed in the amino acid arm, dihydrouracil ring, TφC ring, and anticodon ring. The length of the amino acid arm was 6–7 nt, the dihydropyrimidine ring was 3–5 nt, the TφC ring was 4–6 nt, and the anticodon ring was 3–5 nt. Among the 22 tRNA genes, the *tRNA^Arg^* gene lacked the dihydrouracil ring, and the *tRNA^Phe^* gene lacked the TφC ring (Figure 6).

The relative molecular weight of the rRNA gene is high, and it can combine with proteins to form ribosomes. The lengths of the ribosomal small subunit *rrnS* and ribosome large subunit *rrnL* genes in the mitochondria of *A. lactinea* were 819 and 1396 bp, respectively. *rrnS* was located between the D-loop and *tRNA^Val(GTA)^* gene. *rrnL* was located between *tRNA^Val(GTA)^* and *tRNA^Leu(CTA)^*. The A+T content of *rrnS* and *rrnL* was 85.28%, indicating an obvious AT bias (Figure 1, Table 2 and Table 3).

### 3.5. Control Area, Non-Coding Area, and Overlapping Area

A hypothetical control region (D-loop region) of 303 bp in length was found between *rrnS* and *tRNA^Met^*, with a 74.25% AT content, significant skew towards T between A and T (Table 3), and no tandem repeats in the control region. There were 23 non-coding regions (also known as intergenic spacer regions) in the mitochondrial genome, the lengths of which ranged from 1 to 52 bp, totaling 297 bp. Only two intergenic spacer regions were longer than 40 bp, among which the length of the spacer region between *tRNA^Gln^* and *ND2* was 52 bp. The length of the spacer between *ND5* and *tRNA^His^* was 51 bp. Six genes overlapped, with overlap lengths ranging from 1 to 8 bp. The overlap between the *tRNA^Trp^* and *tRNA^Cys^* genes was the largest, with a length of 8 bp.

### 3.6. Phylogenetic Relationships

**Figure 7:**
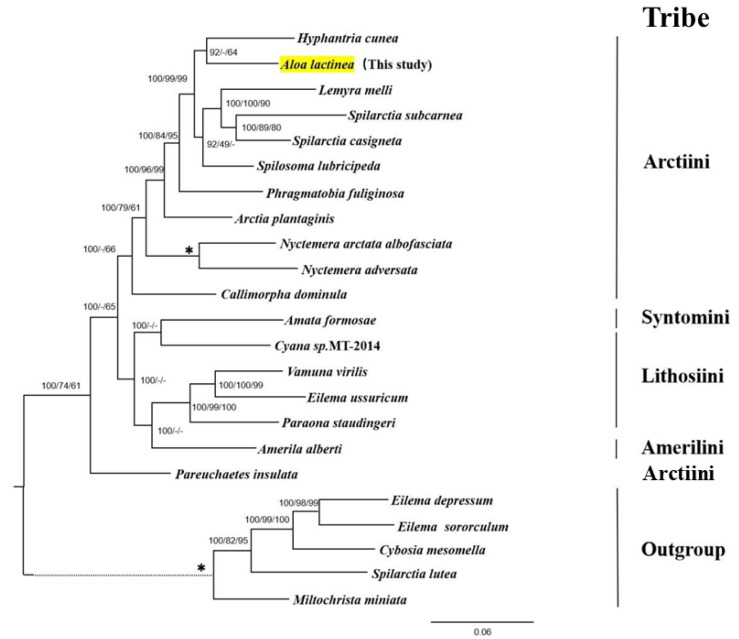
The Bayesian tree based on the complete mitochondrial genome of 23 species in Arctiinae. The phylogenetic tree of *A*. *lactinea* was constructed using Bayesian Inference, Maximum Likelihood, and Maximum Parsimony, all yielding identical topologies. The numbers above the branches are the support rates of Bayesian analysis, maximum likelihood analysis, and maximum reduction analysis, respectively. All nodes with 100% support rates are represented by *, and the evolutionary branch lengths of outgroups are represented by dashed lines.

The whole mitochondrial genome sequences of 23 Arctiinae species were used to construct BI, maximum parsimony, and maximum likelihood phylogenetic trees with the same topology (Figure 7). Among these, *A. lactinea* and *Hyphantria cunea* clustered together (BPP = 100%, MPBP (Maximum parsimony bootstrap proportion) = 64%), indicating that red lactinea and American white moths are closely related. The two *Spilarctia* species were sister groups to *Lemyra* (BPP = 100%, maximum likelihood = 100%, and MPBP = 90%) and *Spilosoma* (BPP = 92% and maximum likelihood = 49%). *Phragmatobia fuliginosa*, *Arctia plantaginis*, *Callimorpha dominula*, and *Pareuchaetes insulata* were also isolated.

Six species of *Amata formosae*, *Cyana* sp. *MT-2014*, and *Amerila alberti* were grouped together into one family. *Amata formosae* and *Cyana* sp. *MT-2014* were clustered together, and the confidence of the Bayesian analysis was 100%, indicating that the two are closely related; however, the branch was not found in the maximum likelihood and maximum parsimony analyses. This group was a sister group to *Paraona*, and all support rates were greater than 95%.

## 4. Discussion

In this study, the mitochondrial whole genome sequence was determined for the first time in this species, and it is also the only species in this genus that has great significance for the study of phylogenetic relationships in this genus. The results indicate that the mitochondrial genome is a circular double-stranded DNA molecule containing 37 genes and one control region, a structure consistent with that of most insects in the order Lepidoptera [13,14]. The mitochondrial genome shows a significant A+T bias, which is a common feature in arthropods [45,46], with the A+T content of all genes being much higher than the G+C content. This nucleotide composition bias is in line with that of other species in the subfamily Arctiinae [14,30,31]. All protein-coding genes begin with the ATN codon, which is different from the COI gene of *Hyphantria cunea* and *Amata* emma in the Arctiinae, where the start codon is CGA [30,31]. This difference may be related to gene rearrangement or editing [47]. The codon usage of all PCGs also exhibits a strong A+T bias, which is a feature shared by other species in the Arctiinae [42,43,44]. All tRNA genes, except for *tRNA^Ser(AGC)^*, form typical cloverleaf structures, which is consistent with the tRNA gene characteristics of other Arctiinae species [42,44]. The control region is located between 12S rRNA and *tRNA^Met^*, with an average length of 439 bp and a very high A+T content (93.5%), which is consistent with the findings of Chen Lu, who analyzed mitochondrial genome structural features of 1116 species from 21 families within the Lepidoptera [14]. Additionally, no tandem repeats were found within the control region, which conforms to the structural characteristics of the control region in Lepidoptera [14,48]. However, the A+T content of this control region is only 74.25%, significantly lower than that of other regions in the mitochondrial genome of Lepidoptera [49], and notably lower than in other species of Arctiinae [30,43].

In this research, we constructed a Bayesian tree of 18 species based on the whole mitochondrial genome sequence, and discussed the relationship between some genera and species of the subfamily. The results show that the relationship between *A. lactinea* and *Hyphantria cunea* is close. The topological structure of the Bayesian tree indicates that the *Spilarctia* and the *Lemyra* are sister groups. The clade formed by these two genera is sister to *Spilosoma*. Similarly, the *Vamuna* and the *Eilema* form a sister group pair, and the clade they constitute is sister to the *Paraona*. This result is consistent with the maximum likelihood tree structure constructed by Galarza and Mappes in 2021 [42]. Furthermore, in this study, *Amata* and *Cyana* cluster into a distinct, well-supported clade in Bayesian analyses, demonstrating a close phylogenetic relationship between them. This finding indirectly suggests that Syntomini is phylogenetically closer to *Cyana* (Lithosiini) than to Arctiini. Pareuchaetes insulata was placed at the base of the Bayesian tree, indicating its relatively primitive position. The subfamily Arctiinae consists of four main tribes: Arctiini, Lithosiini, Syntomini, and Amerilini [9,15,16,17,18]. Since the 1990s, some researchers have argued that Syntomini shares morphological traits with Arctiini (e.g., the presence of a tympanic apparatus on the posterior metathorax and an anterior spiracular hood) and has feeding habits similar to Lithosiini, leading to its classification within Arctiini. However, many scholars have questioned this placement [2,18,50]. Recent molecular studies have provided increasing evidence for including Syntomini within Arctiinae, but its exact tribal placement remains debated. In our study, Syntomini, Lithosiini, and Amerilini formed a clade, supporting a close relationship among them but not confirming the inclusion of Syntomini within Arctiini. Instead, our results suggest that Syntomini is phylogenetically distinct from Arctiini and may warrant separate tribal status within Arctiinae.

## 5. Conclusions

In this study, the complete mitochondrial genome of *A*. *lactinea* was sequenced for the first time, filling a gap in the mitochondrial genomic data for this genus. The results revealed that the mitochondrial genome is a circular double-stranded DNA molecule, 15,380 bp in length, containing 37 genes and one control region, with a high A+T bias characteristic of Lepidoptera mitochondrial genomes. Phylogenetic analysis, based on the complete mitochondrial genomes of 18 species, constructed a Bayesian tree that elucidated the relationships among certain genera and species within the subfamily. The findings support the inclusion of Syntomini within Arctiinae and indicate a close phylogenetic relationship between Syntomini, Lithosiini, and Amerilini. These results provide new molecular evidence for the study of Lepidoptera mitochondrial genomes and their phylogenetic relationships.

## Figures and Tables

**Table 2:** Thirty-seven genes and one control region of the complete mitogenomes in the *A*. *lactinea*.

Gene	Strand	Position	Length	Start Codon	Stop Codon	Anti Codons	Intergenic Nucleotides	Overlapping Nucleotide
*tRNA^Met^*	H	1–67	67	-	-	CAT	-	
*tRNA^Ile^*	H	70–133	64	-	-	GAT	2	
*tRNA^Gln^*	L	131–199	69	-	-	TTG		3
ND2	H	252–1262	1011	ATT	TAA		52	
*tRNA^Trp^*	H	1264–1332	69	-	-	TCA	1	
*tRNA^Cys^*	L	1325–1389	65	-	-	GCA		8
*tRNA^Tyr^*	L	1390–1457	68	-	-	GTA		
COI	H	1495–3000	1506	ATT	TAA		37	
*tRNA^Leu(TTA)^*	H	2996–3062	67	-	-	TAA		5
COII	H	3063–3744	682	ATG	T			
*tRNA^Lys^*	H	3745–3814	70	-	-	CTT		
*tRNA^Asp^*	H	3815–3881	67	-	-	GTC		
ATP8	H	3882–4043	162	ATT	TAA			
ATP6	H	4037–4714	678	ATG	TAA			7
COIII	H	4719–5510	792	ATG	TAA		4	
*tRNA^Gly^*	H	5514–5578	65	-	-	TCC	3	
ND3	H	5588–5932	345	ATA	TAA		9	
*tRNA^Ala^*	H	5932–5999	68	-	-	TGC		1
*tRNA^Arg^*	H	6012–6074	63	-	-	TCG	12	
*tRNA^Asn^*	H	6076–6140	65	-	-	GTT	1	
*tRNA^Ser(AGC)^*	H	6151–6216	66	-	-	GCT	10	
*tRNA^Glu^*	H	6229–6294	66	-	-	TTC	12	
*tRNA^Phe^*	L	6301–6367	67	-	-	GAA	6	
ND5	L	6371–8059	1689	ATA	TAA		3	
*tRNA^His^*	L	8111–8177	67	-	-	GTG	51	
ND4	L	8178–9516	1339	ATG	T			
ND4L	L	9519–9815	297	ATA	TAA		2	
*tRNA^Thr^*	H	9822–9886	65	-	-	TGT	6	
*tRNA^Pro^*	L	9887–9953	67	-	-	TGG		
ND6	H	9961–10,491	531	ATA	TAA		7	
CYTB	H	10,499–11,650	1152	ATG	TAA		7	
*tRNA^Ser(TCA)^*	H	11,680–11,747	68	-	-	TGA	29	
ND1	L	11,765–12,703	939	ATG	TAA		17	
*tRNA^Leu(CTA)^*	L	12,705–12,772	68	-	-	TAG	1	
*18S rRNA*	L	12,795–14,190	1396	-	-		22	
*tRNA^Val^*	L	14,194–14,259	66	-	-	TAC	3	
*12S rRNA*	L	14,259–15,077	819	-	-			1
D-loop	H	15,078–15,380	303	-	-			

**Table 3:** The base composition of the mitochondrial genome of *A. lactinea*.

Gene/Region	Base Composition (%)						AT Skew	GC Skew
	A	T	C	G	A+T	G+C		
Genome	39.95	41.05	11.37	7.62	81.01	18.99	−0.0136	−0.1975
Protein-coding genes (total)	34.48	45.44	9.65	10.44	79.92	20.08	−0.1371	0.0393
ND2	36.30	48.66	9.20	5.84	84.96	15.04	−0.1455	−0.2234
COI	31.54	41.10	13.75	13.61	72.64	27.36	−0.1316	−0.0051
COII	35.78	41.50	12.46	10.26	77.28	22.72	−0.0740	−0.0968
ATP8	45.06	48.77	4.32	1.85	93.83	6.17	−0.0395	−0.4003
ATP6	35.55	43.81	12.83	7.82	79.36	20.65	−0.1041	−0.2426
COIII	33.08	41.54	13.51	11.87	74.62	25.38	−0.1134	−0.0646
ND3	35.65	46.96	11.30	6.09	82.61	17.39	−0.1369	−0.2996
ND5	35.70	46.12	6.10	12.08	81.82	18.18	−0.1274	0.3289
ND4	35.18	47.27	5.90	11.65	82.45	17.55	−0.1466	0.3276
ND4L	31.99	54.55	3.03	10.44	86.54	13.47	−0.2607	0.5501
ND6	37.10	50.09	8.10	4.71	87.19	12.81	−0.1490	−0.2646
CYTB	33.33	44.18	12.67	9.81	77.51	22.48	−0.1400	−0.1272
ND1	31.95	47.28	7.24	13.53	79.23	20.77	−0.1935	0.3028
First site	36.57	45.66	8.41	9.33	82.24	17.75	−0.1105	0.0518
Secondary site	35.06	43.42	10.44	11.06	78.49	21.5	−0.1065	0.0288
Tertiary site	31.75	47.23	10.09	10.92	78.97	21.01	−0.1960	0.0395
tRNA gene	41.38	40.49	7.57	10.57	81.87	18.13	0.0109	0.1655
rRNA gene	43.12	42.17	4.74	9.98	85.28	14.72	0.0111	0.3559
D-loop zone	32.01	42.24	11.88	13.86	74.25	25.75	−0.1378	0.0769

**Table 4:** Comparison of different components of the mitochondrial whole genome in 18 species of Arctiinae.

Species	Whole Genome			PCGs			tRNA Gene			rRNA Gene			D-Loop		
	Size	A+T	GC/AT Skew	Size	A+T	GC/AT Skew	Size	A+T	GC/AT Skew	Size	A+T	GC/AT Skew	Size	A+T	GC/AT Skew
	(bp)	(%)		(bp)	(%)		(bp)	(%)		(bp)	(%)		(bp)	(%)	
*Aloa lactinea*	15,380	81.01	14.522059	11,123	79.92	0.0393/−0.1371	1467	81.87	0.1655/0.0109	2215	85.28	0.3559/0.0111	303	74.25	0.0769/−0.1378
*Vamuna virilis*	15,417	80.4	458.2	10,755	78.14	0.0467/−0.1564	1456	81.59	0.1793/0.0219	2176	84.6	0.3727/0.0310	362	95.03	8.7214397
*Eilema*	15,344	80.46	452.4	11,199	78.75	0.0118/−0.1512	1465	81.91	0.1852/0.0383	2034	84.27	16.299145	329	94.22	1.5237173
*ussuricum*															
*Cyana* sp.	15,494	81.2	15.622378	10,767	79.18	0.0634/−0.1510	1475	81.69	0.1628/0.0224	2192	84.67	0.3810/0.0464	\	\	\
*MT-2014*															
*Paraona staudingeri*	15,427	80.19	−29.17442	10,821	78.03	0.0073/−0.1464	1462	81.12	0.1525/0.0270	2181	84.46	0.3925/0.0000	362	94.48	28.409091
*Spilosoma*	15,369	81.38	13.305732	10,734	79.54	0.0323/−0.1433	1463	81.68	0.1714/0.0159	2187	85.05	0.3271/0.0140	361	95.01	4.8993363
*lubricipeda*															
*Arctia plantaginis*	15,479	80.78	14.15528	10,731	78.7	0.0413/−0.1422	1464	81.56	0.1779/0.0267	2204	84.53	18.743719	401	96.01	8.2881806
*Hyphantria cunea*	15,481	80.38	−23.75258	10,752	78.18	0.0339/−0.1480	1473	81.74	0.1599/0.0250	2212	84.67	0.3509/0.0017	357	94.96	14.631579
*Callimorpha dominula*	15,496	81.02	18.46789	10,785	79.85	0.0491/−0.1456	1462	82.08	0.1607/0.0217	2154	84.54	15.012195	486	75.1	−2.231746
*Lemyra melli*	15,418	78.67	−204.1818	10,749	76.2	0.0361/−0.1512	1468	80.65	0.1829/0.0068	2233	84.19	−117.5938	338	94.38	5.5945626
*Nyctemera*	15,432	80.05	15.489796	10,761	78.94	0.0228/−0.1430	1445	81.11	0.1941/0.0359	2058	84.25	13.5	\	\	\
*albofasciata*															
*Nyctemera*	15,429	79.21	2670	10,761	76.67	0.0212014	1460	81.16	0.1783/0.0261	2033	84.55	14.264	292	94.86	129.69444
*adversata*															
*Phragmatobia fuliginosa*	15,469	80.98	26.785714	10,767	78.88	0.0208/−0.1496	1463	81.68	0.1567/0.0260	2181	85.1	0.3597/0.0172	181	96.69	0.0000/−0.0057

**Table 5:** Statistics of codon usage in the *A. lactinea*.

Codon	Amino Acid	*n*	%	RSCU	Codon	Amino Acid	*n*	%	RSCU
GCA	Ala	48	1.30	1.51	**AAA**	Lys	98	2.65	1.90
GCC	Ala	3	0.08	0.09	AAG	Lys	5	0.14	0.10
GCG	Ala	2	0.05	0.06	**AUA**	Met	277	7.49	1.87
**GCU**	Ala	74	2.00	2.33	AUG	Met	20	0.54	0.13
**CGA**	Arg	39	1.06	3.00	UUC	Phe	21	0.57	0.12
CGC	Arg	0	0.00	0.00	**UUU**	Phe	323	8.74	1.88
CGG	Arg	2	0.05	0.15	CCA	Pro	31	0.84	0.98
CGU	Arg	11	0.30	0.85	CCC	Pro	7	0.19	0.22
AAC	Asn	10	0.27	0.08	CCG	Pro	2	0.05	0.06
**AAU**	Asn	238	6.44	1.92	CCU	Pro	86	2.33	2.73
GAC	Asp	1	0.03	0.03	**AGA**	Ser	88	2.38	2.23
**GAU**	Asp	62	1.68	1.97	AGC	Ser	0	0.00	0.00
UGC	Cys	0	0.00	0.00	AGG	Ser	0	0.00	0.00
**UGU**	Cys	29	0.78	2.00	AGU	Ser	26	0.70	0.66
**CAA**	Gln	62	1.68	2.00	UCA	Ser	78	2.11	1.98
CAG	Gln	0	0.00	0.00	UCC	Ser	9	0.24	0.23
**GAA**	Glu	73	1.98	1.90	UCG	Ser	0	0.00	0.00
GAG	Glu	4	0.11	0.10	**UCU**	Ser	114	3.08	2.90
**GGA**	Gly	111	3.00	2.24	ACA	Thr	61	1.65	1.64
GGC	Gly	0	0.00	0.00	ACC	Thr	14	0.38	0.37
GGG	Gly	25	0.68	0.51	ACG	Thr	1	0.03	0.03
GGU	Gly	62	1.68	1.25	**ACU**	Thr	73	1.98	1.96
CAC	His	9	0.24	0.28	UGG	Trp	1	0.03	0.02
**CAU**	His	56	1.52	1.72	**UGA**	Trp	95	2.57	1.98
AUC	Ile	16	0.43	0.07	UAC	Tyr	10	0.27	0.10
**AUU**	Ile	451	12.2	1.93	**UAU**	Tyr	187	5.06	1.90
CUA	Leu	16	0.43	0.18	**GUA**	Val	68	1.84	2.05
CUC	Leu	2	0.05	0.02	GUC	Val	2	0.05	0.06
CUG	Leu	0	0.00	0.00	GUG	Val	0	0.00	0.00
CUU	Leu	43	1.16	0.47	GUU	Val	63	1.70	1.89
**UUA**	Leu	475	12.85	5.20	UAG *	-	-	-	-
UUG	Leu	12	0.32	0.13	UAA *	-	-	-	-

A total of 3696 codons were analyzed. RSCU represents the frequency of synonymous relative codons. Bold type indicates the most frequently used codon in a single amino acid. * indicates a stop codon.

## Data Availability

The data presented in this study are available in NCBI GenBank (Accession number: PV288325).
